# Ecological and Management Drivers of Population Fluctuations in the Goitered Gazelle ( *Gazella subgutturosa* ) Across Iran

**DOI:** 10.1002/ece3.74234

**Published:** 2026-08-31

**Authors:** Mohammad Sepehri, Hossein Varasteh Moradi, Hamid‐Reza Rezaei, Mahmoud‐Reza Hemami

**Affiliations:** ^1^ Environmental Sciences Department, Faculty of Fisheries and Environmental Sciences Gorgan University of Agricultural Sciences and Natural Resources Gorgan Iran; ^2^ Department of Natural Resources Isfahan University of Technology Isfahan Iran

**Keywords:** arid and semi‐arid ecosystems, goitered gazelle, human land‐use, Iran, management effectiveness, population dynamics, principal component analysis, protected areas

## Abstract

Understanding the ecological and anthropogenic determinants of ungulate population dynamics is essential for conservation in arid and semi‐arid ecosystems. We analysed 16 years (2007–2022) of census data from 27 protected areas across Iran to identify climatic, human land‐use, and management factors influencing population growth of the goitered gazelle ( *Gazella subgutturosa* ). Annual exponential growth rates were estimated by regressing the natural logarithm of population size against year. The best‐supported model explained 46% of the variation in population growth (*R* ^2^ = 0.46, adjusted *R* ^2^ = 0.41). Population growth was negatively associated with a protection component reflecting longer protection history, higher protection status, and greater management infrastructure, consistent with density‐dependent regulation in established reserves. Growth was positively associated with a human land‐use component describing wells and springs in surrounding landscapes, suggesting that water availability beyond reserve boundaries may contribute to greater resource availability. Climatic principal components and protected‐area classes representing broad climatic–productivity regimes were not detected as independent predictors of population growth within the analytical framework and spatial and temporal scales examined. These results highlight the importance of water availability, management legacy, and landscape context for understanding population dynamics and informing adaptive, landscape‐scale conservation planning.

## Graphical abstract

We analysed 16 years of census data from 27 protected areas across Iran to identify factors associated with population growth in the goitered gazelle ( *Gazella subgutturosa* ). Population dynamics were associated primarily with protection history and management characteristics and with water availability in surrounding landscapes, while broad‐scale climatic gradients showed no detectable independent association at the spatial and temporal scales examined. These results highlight the importance of adaptive management and landscape‐level planning for conserving large herbivores in arid and semi‐arid ecosystems.

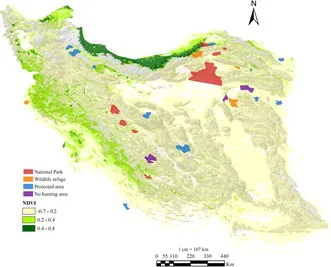

## 1 Introduction

Identifying ecological and anthropogenic drivers of population change in large herbivores is central to biodiversity conservation, particularly in arid and semi‐arid regions where water scarcity, climatic variability, and expanding human activities shape wildlife dynamics. Ungulates play fundamental ecological roles by regulating vegetation dynamics, facilitating nutrient cycling, and sustaining predator populations (Danell et al. 2006). However, many ungulate species are declining due to intensified climate pressures and human disturbance, underscoring the need to understand the factors governing their persistence (Ripple et al. 2015; Tsindi et al. 2016).

The goitered gazelle ( *Gazella subgutturosa* ) is one of the dominant native herbivores of Iran's plains and steppes and constitutes a principal prey species for the Critically Endangered Asiatic cheetah ( *Acinonyx jubatus venaticus* ), linking its conservation directly to the survival of Iran's top predator. Historically, the species ranged widely from the Zagros foothills through Central Asia to Mongolia, China, and Pakistan (Hemami et al. 2020). Over the past century, however, populations have declined sharply due to poaching, overgrazing, and habitat degradation (Hemami and Groves 2001). Population trajectories vary considerably across Iran. For example, some reserves have experienced substantial declines during the last decade whereas others have shown rapid population increases, suggesting that demographic responses are strongly context dependent. These contrasting trends indicate that population dynamics are governed by multiple, site‐specific drivers that require integrated assessment. Population growth ultimately reflects the balance among recruitment, survival, immigration, and emigration, all of which may respond to resource availability, population density, and environmental conditions (Caughley 1994; Bonenfant et al. 2009).

Climatic variability strongly influences ungulate demography by shaping primary productivity, water availability, and forage quality. From a resource‐limitation perspective, water availability is a fundamental determinant of herbivore population dynamics in dryland ecosystems. In arid and semi‐arid environments such as much of Iran, precipitation regulates primary productivity, forage quantity, and forage quality, thereby constraining carrying capacity and population growth (Illius and O'Connor 2000). Consequently, herbivore populations may respond strongly to spatial and temporal variation in water‐dependent resources. Rainfall and temperature fluctuations can generate marked demographic responses over both short and multi‐annual periods (DeMars et al. 2021), while vegetation indices such as the Normalised Difference Vegetation Index (NDVI) may capture ecologically relevant variation in herbivore abundance and growth more effectively than rainfall alone (Ogutu et al. 2008; Pettorelli et al. 2011). Water availability may also directly influence herbivore distribution and habitat use through its effects on forage accessibility and plant quality, particularly in water‐limited ecosystems (Henley et al. 2007).

Disturbance ecology provides an additional framework for understanding population dynamics in dryland systems. Human‐induced disturbances such as livestock grazing, agricultural expansion, infrastructure development, and groundwater extraction can alter vegetation structure, reduce habitat quality, and disrupt access to key resources. These effects may interact with climatic stressors, amplifying ecological degradation and reducing ecosystem resilience (Chaturvedi et al. 2021). Human activities can also fragment habitats and increase mortality risks through poaching and other forms of human disturbance (Ripple et al. 2015). Protected‐area management can mitigate some of these pressures through law enforcement, habitat management, regulation of human activities, and local community engagement (Kragt et al. 2020; Kuiper et al. 2021). Yet, management intensity may also respond to population status, as areas supporting larger or more vulnerable populations may receive greater conservation investment. Quantifying the relative contributions of management, human disturbance, and climate is therefore crucial for identifying conditions that support resilient gazelle populations.

Despite the ecological importance of the goitered gazelle, no nationwide assessment has examined how climatic, anthropogenic, and management factors jointly influence its population dynamics in Iran. A national‐scale perspective is particularly important in Iran because protected areas span pronounced gradients in climate, water availability, human pressure, and management history. Relationships observed at individual sites may therefore not be representative of the broader processes governing gazelle populations across the country. Previous studies have generally been site‐specific or short‐term and have not examined interactions between management practices and human pressures (Nowzari et al. 2007; Hazeri et al. 2009; Farhadinia et al. 2009; Almasieh and Mohammadi 2023). To fill this gap, we conducted the first national‐scale analysis, integrating 16 years (2007–2022) of systematic census data from 27 protected areas with spatial variation in protection history, enforcement capacity, and human land use. We combined principal component analysis (PCA) to summarise correlated climatic, human land‐use, and protection‐related variables with General Linear Models (GLMs) to evaluate their associations with population growth.

Drawing on resource‐limitation theory, disturbance ecology, and density‐dependent population regulation, we hypothesised that (1) population growth would increase in protected areas characterised by greater water availability and accessible habitats; (2) long‐established reserves with higher protection levels and management investment would exhibit lower exponential growth rates, potentially reflecting density‐dependent regulation in populations approaching ecological limits; and (3) human land‐use variables would be associated with gazelle population growth, with resource‐related variables such as water availability potentially showing positive associations and disturbance‐related variables showing negative associations.

## 2 Methods

### 2.1 Study Area and Census Data

**FIGURE 1:**
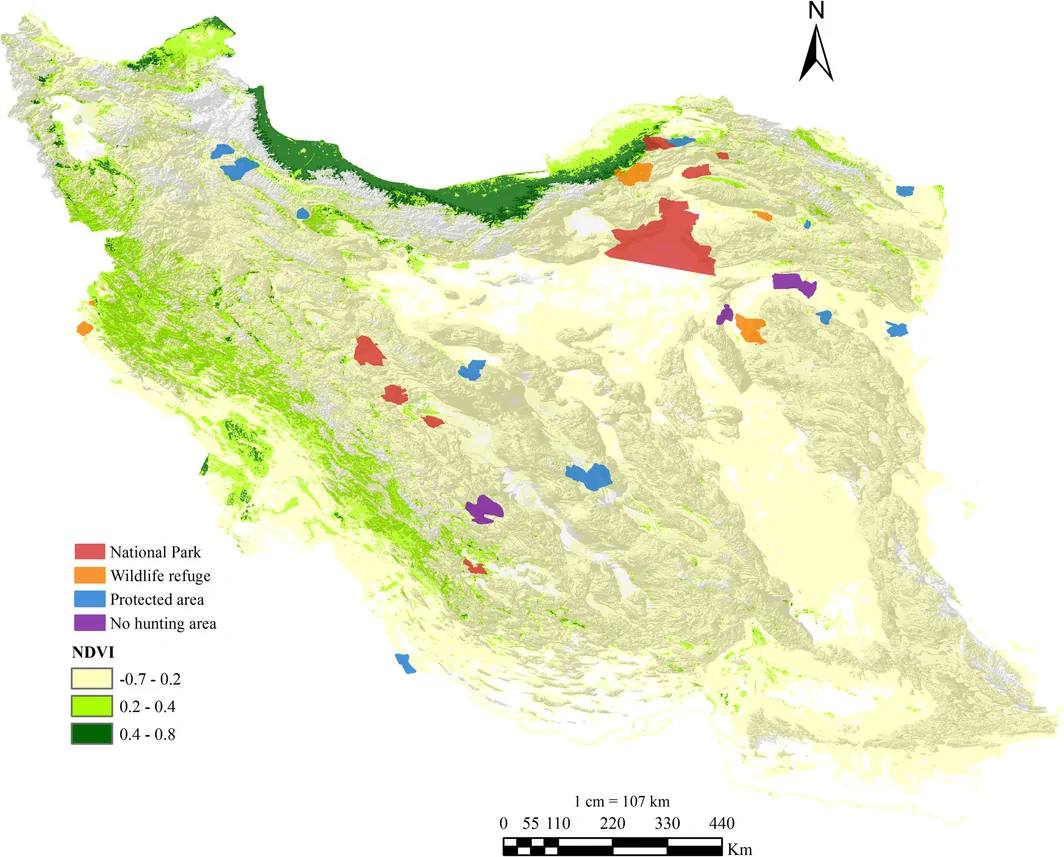
Spatial distribution of the 27 protected areas supporting goitered gazelle populations across Iran. Protected areas are shown according to conservation designation, overlaid on mean NDVI and hillshade layers representing vegetation productivity and topographic heterogeneity, respectively. The map illustrates the broad environmental variation across the study sites and the distribution of gazelle populations within predominantly arid and semi‐arid landscapes.

The goitered gazelle occurs throughout Iran's extensive arid and semi‐arid steppes, which extend from cold northeastern plains to hot southwestern lowlands. Much of this distribution lies within Iran's extensive drylands and steppes, which together account for approximately 85% of the country's land area. Within the study area, comprising 27 protected areas, mean annual precipitation ranges from approximately 55 mm in central desert regions to more than 370 mm in northern habitats, while temperatures vary from minimum values of about −8°C to maximum values exceeding 47°C. Vegetation across these landscapes is dominated by drought‐adapted species, particularly *Artemisia* spp. and *Astragalus* spp. The grey wolf ( *Canis lupus* ) is the main predator of the gazelle, while the cheetah and caracal ( *Caracal caracal* ) also occur in some protected areas and occasionally prey on it. Annual census data from 27 protected areas were obtained from the Iranian Department of Environment for the period 2007 to 2022. Population counts were derived from annual direct‐count surveys conducted by trained personnel of the Iranian Department of Environment using standardised monitoring protocols. Although observer teams and local survey conditions could vary among protected areas and years, the same general census protocol was applied throughout the monitoring period, and no systematic changes in census methodology were identified in the records used for this study. Population counts were not corrected for detection probability because comparable detectability estimates were unavailable across all protected areas. Consequently, census counts were treated as indices of relative population size rather than absolute abundance. Although annual counts are subject to observation error and census uncertainty, the use of a 16‐year time series and the broad consistency of survey methods within protected areas are expected to reduce the influence of short‐term counting errors on estimates of long‐term population growth rates. The spatial distribution of these protected areas across Iran is shown in Figure 1. These areas were selected from an initial set of 66 based primarily on the availability of complete and consistent long‐term census records. Areas with substantial gaps in census records, major changes in survey methodology, or monitoring periods shorter than 10 years were excluded. The selected sites nevertheless encompassed the major climatic and ecological conditions represented in the species' distribution across Iran. According to the national ecological classification of Iran, the study sites represent the major dryland biomes occupied by goitered gazelles, including desert plains, semi‐desert shrublands, and Artemisia‐dominated steppes. For each protected area, the exponential population growth rate (*r*) was estimated by regressing the natural logarithm of the population size against year. The slope of this regression provided an estimate of the average annual exponential growth rate over the 16‐year study period, and was used as the dependent variable in subsequent analyses. Because population growth rate was estimated as a single long‐term parameter for each protected area from the 2007–2022 census series, the final regression analysis contained one response value per site and therefore did not involve repeated annual observations. Temporal autocorrelation was consequently not applicable to the final cross‐sectional model.

We acknowledge that restricting analyses to protected areas with complete long‐term monitoring records may introduce some sampling bias toward better‐monitored reserves. However, the selected sites span a wide range of environmental conditions, reserve sizes, and management contexts, reducing the likelihood that results are driven solely by monitoring quality.

### 2.2 Predictor Variables

Predictor variables were organised into three categories representing climatic conditions, human land use, and protection/management characteristics. Climatic variables included mean annual temperature; mean maximum temperatures for June, July, August, and September; mean minimum temperatures for December, January, and February; mean annual rainfall; mean rainfall for March, April, and May; and the mean annual number of rainy days, all calculated over the 16‐year study period. In addition, mean NDVI values over the same period and temporal trends in spring NDVI were derived from MODIS 250 m seasonal imagery as indicators of vegetation productivity and dynamics.

Human land‐use variables captured both on‐site pressures and influences from surrounding landscapes. These included the number of villages, agricultural fields, mines, and other human land uses within protected areas, as well as the total length of asphalt and unpaved roads. Variables describing surrounding land use were quantified within a 15 km buffer zone and included the number of wells, springs, villages, towns or cities, and industrial facilities. Indicators of human presence and disturbance included livestock grazing duration and an anthropogenic fatality rate, calculated as the combined number of poaching incidents and traffic‐related mortalities expressed as a percentage of the population size.

Protection and management variables described both structural and operational aspects of reserve management. These included the extent of potential gazelle habitat within each protected area, defined as terrain with slopes below 30%; the edge‐to‐area ratio; an isolation index defined as the mean distance to the three nearest protected areas; relative staffing levels; relative numbers of patrol vehicles and guard posts; the density of natural and artificial water points; protection level; and the number of years since initial designation. Data were compiled from Iranian Department of Environment administrative records and verified through questionnaires completed by reserve managers.

### 2.3 Analyses

PCA was used to reduce dimensionality and multicollinearity within the climatic, human land‐use, and management datasets. The analysis included quantitative variables measured on ratio or interval scales, encompassing both continuous variables (e.g., climatic metrics and NDVI) and count‐based indicators of infrastructure and management (e.g., number of farms, mines, and villages). Separate PCAs were conducted for climatic, human land‐use, and protection/management variables because these datasets represented distinct ecological processes and contained correlated predictors. Analysing them separately improved interpretation of the resulting components and reduced the potential for variable groups containing larger numbers of correlated predictors to dominate the ordination. All variables were standardised prior to analysis to account for differences in measurement units and variance, an approach commonly adopted when applying PCA to heterogeneous ecological datasets (Legendre and Legendre 2012; Borcard et al. 2011). Components with eigenvalues greater than one were retained, with this selection further supported by inspection of scree plots. Component loadings were examined to identify the dominant ecological and management gradients represented by each axis.

To account for broad‐scale environmental heterogeneity among protected areas, we applied a k‐means cluster analysis to long‐term climatic variables and seasonal NDVI, grouping the 27 protected areas into two discrete classes representing major climatic–productivity regimes (Table S1). The population growth rate (*r*) was analysed using General Linear Models implemented as multiple linear regression. PCA scores derived from climatic, protection/management, and human land‐use variables were entered as continuous predictors. Protected‐area class was included as a categorical predictor (dummy coded) to test for additional effects of broad‐scale climatic–productivity differences beyond those captured by the PCA axes.

An initial full multiple linear regression model included all retained PCA components together with protected‐area class. Backward elimination was used as an exploratory model‐simplification procedure to obtain a parsimonious subset of predictors, while competing models were evaluated using Akaike's Information Criterion (AIC), Bayesian Information Criterion (BIC), adjusted *R* ^2^, and ecological interpretability. Given the relatively small sample size and the large initial set of PCA‐derived predictors, this approach provided a pragmatic means of reducing model complexity while retaining ecological interpretability. Protected‐area class did not improve model fit and was therefore excluded from the final model, which retained Protection Component 2 and Human Land‐use Component 3. Protected‐area class did not improve model fit and was therefore excluded from the final model, which retained Protection Component 2 and Human Land‐use Component 3.

Model assumptions were evaluated through visual inspection of standardised residuals versus fitted values and Q–Q plots, which indicated homoscedasticity and approximate normality of residuals. Linearity between predictors and the response variable was assessed through inspection of residual‐versus‐fitted plots and partial regression plots, with no substantial departures from linearity detected. Multicollinearity among predictors retained in the final model was assessed using Variance Inflation Factors (VIF) and tolerance statistics; all VIF values were below 1.005, indicating negligible collinearity among predictors. No substantial departures from linearity were detected. Spatial independence of model residuals was assessed using post hoc Moran's *I* tests based on the geographic coordinates of gazelle habitats within the 27 protected areas, with k‐nearest‐neighbour spatial weights (*k* = 3–5), with significance evaluated using 9999 permutations.

## 3 Results

The PCA of climatic variables yielded three components with eigenvalues greater than one, explaining 89.8% of the total variance (Table 1). These components represented major gradients in thermal conditions, moisture availability, and vegetation productivity, and temporal vegetation dynamics. Overall, climatic variation among protected areas was structured primarily by differences in temperature regimes, followed by precipitation–productivity gradients and secondary variation in vegetation dynamics (Table S2).

**TABLE 1:** Principal component analysis (PCA) of climatic and vegetation‐productivity variables across 27 protected areas in Iran.

Component	Eigenvalue	Variance explained (%)	Cumulative variance (%)	Major loadings (> |0.5|)
1	7.156	47.703	47.703	Mean annual temperature; Maximum temperature (June–September); Minimum temperature (December–February)
2	4.905	32.700	80.403	Annual rainfall; Rainfall (March–May); Annual rainy days; 16‐year mean NDVI
3	1.412	9.415	89.818	Spring NDVI change trend

*Note:* Components with eigenvalues > 1 are shown. Major loadings represent variables with absolute values > |0.50|.

The PCA of human land‐use and infrastructure variables produced four components explaining 71.8% of the variance (Table 2). These components reflected gradients of anthropogenic development intensity, urban and industrial activity, water‐resource availability in surrounding landscapes, and transportation accessibility. Together, they captured major differences in the intensity and spatial distribution of human activities within and around protected areas (Table S3).

**TABLE 2:** Principal component analysis (PCA) of human land‐use and infrastructure variables across 27 protected areas in Iran.

Component	Eigenvalue	Variance explained (%)	Cumulative variance (%)	Major loadings (> |0.5|)
1	4.515	34.728	34.728	Farms; Mines; Other human land uses; Villages (inside and buffer); Cities in buffer; Grazing duration; Paved and unpaved road length
2	2.130	16.388	51.117	Cities in buffer; Industrial facilities in buffer; Grazing duration (negative loading)
3	1.443	11.098	62.215	Wells in buffer; Springs in buffer
4	1.253	9.635	71.850	Paved and unpaved road length (surface accessibility gradient)

*Note:* Components with eigenvalues > 1 are shown. Major loadings represent variables with absolute values > |0.50|.

The PCA of protection and management variables yielded three components explaining 77.1% of the variance (Table 3). These components described variation in management and enforcement capacity, protection history and legal status, and reserve configuration. Overall, differences among protected areas were driven primarily by management capacity, followed by protection history and landscape structure (Table S4).

**TABLE 3:** Principal component analysis (PCA) of protection and management variables across 27 protected areas in Iran.

Component	Eigenvalue	Variance explained (%)	Cumulative variance (%)	Major loadings (> |0.5|)
1	4.001	40.005	40.005	Ranger density; patrol vehicle density; guard post density; artificial water‐point density; natural water‐source density
2	2.267	22.666	62.672	Protection duration; protection level; edge complexity (negative loading)
3	1.443	14.433	77.104	Protection level; edge length; plain area (moderate loading)

*Note:* Components with eigenvalues > 1 are shown. Major loadings represent variables with absolute values > |0.50|.

The final multiple regression model relating population growth rate to protection and human land‐use predictors yielded a significant overall fit (*F*_2,24_ = 10.08, *p* = 0.001), explaining 45.6% of the variance in population growth (*R* ^2^ = 0.46; *adjusted R* ^2^ = 0.41) (Table 4). Protection Component 2 showed a significant negative association with population growth (*F*_1,24_ = 12.47, *p* = 0.002), indicating that protected areas characterised by longer protection duration, higher protection status, and greater management infrastructure tended to exhibit lower growth rates. A one‐standard‐deviation increase in Protection Component 2 was associated with an estimated 0.042 decrease in the annual population growth rate (*r*).

**TABLE 4:** Results of the minimal GLM relating population growth rate (*r*) to protection and human‐land‐use predictors across 27 protected areas in Iran.

	*F*_df_	*β* ± SE	*p*	95% CI
Model	10.08_2,24_		0.001	
Intercept	31.77	0.065 ± 0.012	< 0.001	0.041–0.089
Protection C2	12.47_1,24_	−0.042 ± 0.012	0.002	−0.066, −0.017
Human C3	6.38_1,24_	0.030 ± 0.012	0.019	0.005, 0.054

*Note:* Model fit: *R* ^2^ = 0.46, adjusted *R* ^2^ = 0.41.

**FIGURE 2:**
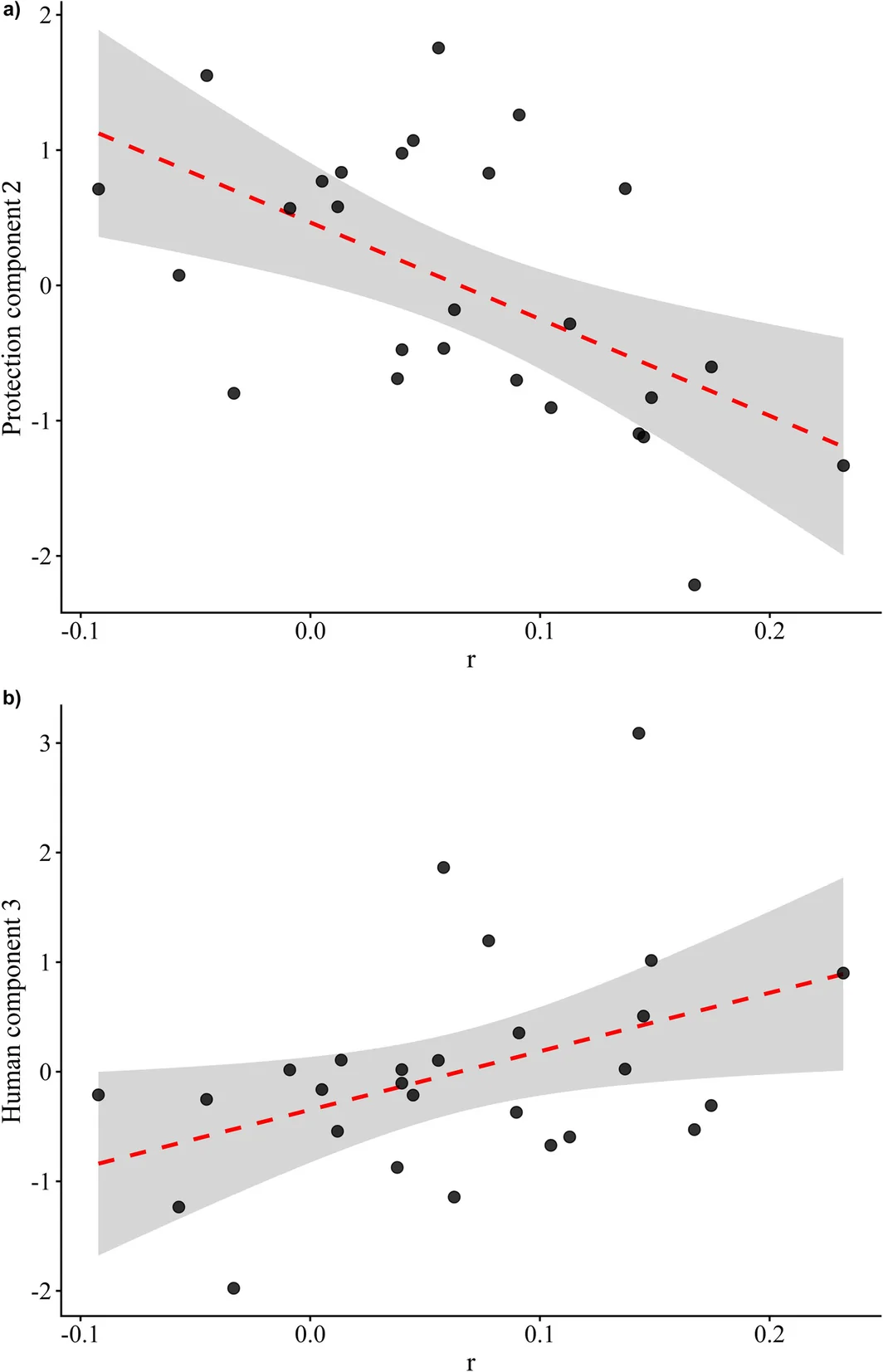
Relationships between population growth rate (*r*) and the significant PCA‐derived predictors: (a) Protection Component 2 and (b) Human Component 3. Dashed lines represent fitted linear regressions and shaded areas indicate 95% confidence intervals. The negative relationship in panel (a) indicates lower population growth rates along the protection‐related gradient, whereas the positive relationship in panel (b) indicates higher growth rates along the gradient associated with greater availability of wells and springs in surrounding landscapes. Regression coefficients were −0.042 ± 0.012 (*β* ± SE) for Protection Component 2 and 0.030 ± 0.012 for Human Component 3. The axes represent standardised PCA component scores. *N* = 27; adjusted *R* ^2^ = 0.41.

Population growth was positively associated with human Land‐use Component 3 (*F*_1,24_ = 6.38, *p* = 0.019), a gradient characterised primarily by the availability of wells and springs in surrounding landscapes. This association may indicate that water availability in surrounding landscapes contributes to resource availability for gazelles. A one‐standard‐deviation increase in Human Component 3 was associated with an estimated 0.030 increase in the annual population growth rate (*r*). This positive relationship suggests that greater water availability in surrounding landscapes may provide supplementary water and forage resources, potentially supporting higher population growth rates (Figure 2).

None of the climatic PCA components was retained in the supported final model. In the full model, none showed a significant association with population growth (*F* ≤ 0.99, *p* ≥ 0.335), indicating that broad‐scale climatic gradients did not have a detectable independent association with population growth at the spatial and temporal scales examined. Diagnostic evaluation indicated that the model assumptions were adequately met: residual plots showed no evidence of substantial heteroscedasticity or deviation from normality, and VIFs were low (< 1.005), indicating negligible multicollinearity among predictors. Model comparisons using Akaike's and Bayesian Information Criteria further supported the reduced model as the most parsimonious representation of the data. Moran's *I* tests provided no significant evidence of spatial autocorrelation in model residuals across the three spatial‐neighbourhood specifications (*k* = 3–5; permutation‐based two‐sided *p* = 0.057–0.110).

## 4 Discussion

This study provides the first nationwide, integrative assessment of ecological, anthropogenic, and management drivers influencing the population dynamics of the goitered gazelle across Iran's protected‐area network. By combining 16 years of systematic census data with spatially explicit climatic, land‐use, and management indicators, the analysis indicates that variation in population growth was most consistently associated with protection‐related structure and human landscape influences. In contrast, climatic PCA components and broad environmental classes were not retained as significant predictors in the final models once management and landscape variables were considered. This result should not be interpreted as evidence that climate is unimportant for gazelle populations, but rather that its independent effect was not detectable at the spatial and temporal scales analysed in this study. Comparable global syntheses have shown that variation in management effectiveness and human pressure often explains wildlife population trends within protected areas more strongly than environmental variables alone (Watson et al. 2014; Barnes et al. 2016; Geldmann et al. 2018; Pulido‐Chadid et al. 2025). The present findings extend this pattern to a wide‐ranging arid‐land ungulate at a national scale, highlighting the potential importance of management configuration and surrounding landscape context in shaping demographic trajectories. The negative association between Protection Component 2 and population growth suggests that protected areas characterised by longer protection history, higher protection status, and greater management infrastructure tend to exhibit lower growth rates. This pattern is consistent with ecological expectations for long‐established reserves in which ungulate populations may have approached local carrying capacity. If populations in these reserves have approached local carrying capacity, density‐dependent processes could reduce recruitment and slow population growth despite high levels of conservation investment. Such density‐dependent responses are widely documented in large herbivores and represent a common regulatory mechanism influencing demographic performance in protected populations (Eberhardt 2002; Bonenfant et al. 2009; Jaggi et al. 2024). However, density dependence was not directly tested in this study because density estimates were not included as predictors and demographic rates such as recruitment and mortality were unavailable. The observed negative association should therefore be interpreted as a plausible ecological explanation rather than direct evidence of density‐dependent regulation. These findings emphasise that management effectiveness should be evaluated not solely through infrastructure or enforcement indicators but also in relation to ecological carrying capacity and population status. Conversely, management investment may itself respond to population status, with greater resources directed toward areas where gazelle populations are declining or considered conservation priorities, so reverse causation cannot be excluded.

In contrast, Human Component 3, representing the density of wells and springs in surrounding landscapes, was positively associated with population growth. Anthropogenic and natural water sources within buffer zones likely enhance forage productivity and expand accessible habitat beyond reserve boundaries. Water availability is widely recognised as a fundamental constraint on distribution, movement, and demographic performance in arid and semi‐arid ungulates (Payne et al. 2020; Schmied née Stommel et al. 2025). In this context, groundwater‐supported springs and human‐developed wells may act as resource subsidies, potentially increasing the amount of accessible forage and water available to gazelles at the landscape scale. Similar dynamics have been observed in African and Asian herbivore systems, where artificial water provision and agricultural edges alter spatial distribution and locally enhance herbivore abundance (Smit et al. 2007; Schmied née Stommel et al. 2025). The findings suggest that demographic processes within protected areas are partly influenced by ecological conditions extending into adjacent multi‐use landscapes.

However, resource subsidies in human‐modified landscapes may simultaneously elevate risks, including poaching, disturbance, disease transmission, and vehicle collisions. Anthropogenic habitats can function as ecological traps if short‐term resource benefits are offset by higher mortality (Valeix et al. 2008; Martinez et al. 2024; Chemwa et al. 2026). The positive association observed here therefore underscores the importance of coordinated landscape‐scale planning. Management strategies should balance the demographic benefits of supplementary water availability with proactive mitigation of anthropogenic threats in buffer zones and surrounding lands.

Climatic PCA components were not retained in the supported models, despite the well‐documented sensitivity of arid‐zone herbivores to rainfall variability and temperature extremes (Illius and O'Connor 2000; Ogutu et al. 2008). Several explanations may account for this pattern. First, the 16‐year monitoring period may not have encompassed sufficiently severe or prolonged climatic anomalies to generate strong demographic contrasts among protected areas. Second, widespread artificial water provision and active management may buffer gazelle populations against short‐term climatic variability. In addition, species inhabiting arid environments often exhibit behavioural and ecological strategies that reduce exposure to resource limitation across environmental gradients, potentially weakening direct climate–population relationships at broad spatial scales (Chaturvedi et al. 2021). Third, the climatic variables used in the analysis primarily represented long‐term or aggregated conditions and may not adequately capture interannual climatic variability or lagged climatic effects, which can be important drivers of demographic fluctuations in dryland systems. A further explanation may involve a scale mismatch between ecological processes and predictor variables. Gazelle demographic responses may be influenced by localised resource conditions or short‐term climatic events that are not adequately represented by protected‐area‐level averages derived from broad‐scale climatic datasets. Furthermore, climatic effects may operate indirectly through vegetation productivity and forage availability rather than through climatic averages themselves. Remote‐sensing indicators such as vegetation indices have often been shown to capture ecologically meaningful responses of herbivores to environmental variability more effectively than long‐term climatic means (Pettorelli et al. 2011). These results suggest that, within the climatic range observed during the study period, demographic differences were more strongly associated with management configuration and landscape resource availability than with broad climatic gradients per se.

In conclusion, the model explained a moderate proportion of variation in population growth (*R* ^2^ ≈ 0.46), indicating that additional unmeasured factors, including predator dynamics, disease, fine‐scale habitat quality and forage composition, livestock competition, undiscovered illegal offtake, and stochastic demographic variation, may also contribute to residual heterogeneity. Nonetheless, the consistent retention of protection history and water‐related landscape variables across model comparisons (including AIC and BIC) supports the robustness of their association with demographic performance. These findings suggest that conservation outcomes in arid protected areas are strongly associated with interactions among management legacy, hydrological resources, and landscape context, while potential climatic influences may operate through indirect pathways or at temporal and spatial scales not captured in the present analysis. From a management perspective, the results highlight the importance of adaptive strategies tailored to population status. In reserves where gazelle populations may be approaching ecological limits, expanding enforcement infrastructure alone may not yield further demographic gains. Instead, emphasis should shift toward maintaining habitat quality, preserving connectivity among reserves, and facilitating natural dispersal. At the same time, strategic planning of natural and artificial water sources is essential. Water distribution influences spatial aggregation, vegetation dynamics, and exposure to risk; thus, its management should integrate ecological objectives with human–wildlife conflict mitigation. Such landscape‐integrated approaches are increasingly recognised as critical for sustaining wide‐ranging herbivores in human‐dominated drylands (Tucker et al. 2018).

## 5 Conclusion

The results of this study indicate that variation in goitered gazelle population growth in Iran is associated with management and landscape factors rather than on protection status alone. In particular, water availability and the configuration of surrounding land uses are associated with variation in population growth. Conservation outcomes can be improved by prioritising the protection and sustainable management of natural water sources, alongside careful regulation and planning of artificial water provisioning in buffer zones. Strengthening coordination between protected‐area authorities and surrounding land users is also essential to reduce external pressures that may compromise habitat quality.

These findings support a shift toward adaptive, landscape‐based management approaches in arid ecosystems, where ecological conditions and resource distribution are highly variable. Enhancing habitat connectivity and integrating cross‐boundary planning frameworks should be considered key priorities for maintaining long‐term population stability. As an observational analysis based on long‐term population indices, future studies should incorporate annual climatic variability, demographic rates, habitat connectivity, and additional ecological drivers to better resolve the mechanisms underlying population dynamics. Continued ecological monitoring combined with spatially explicit management planning and targeted field‐based assessments of resource distribution will be critical for evaluating conservation effectiveness and supporting the long‐term persistence of the species under increasing environmental and anthropogenic pressures.

## Author Contributions

**Mohammad Sepehri:** conceptualization (equal), data curation (lead), formal analysis (equal), visualization (lead), writing – original draft (equal), writing – review and editing (equal). **Hossein Varasteh Moradi:** conceptualization (equal), formal analysis (supporting), validation (equal), writing – review and editing (equal). **Hamid‐Reza Rezaei:** validation (equal), writing – review and editing (equal). **Mahmoud‐Reza Hemami:** conceptualization (lead), formal analysis (equal), methodology (lead), writing – original draft (equal), writing – review and editing (equal).

## Funding

The authors have nothing to report.

## Conflicts of Interest

The authors declare no conflicts of interest.

## Supporting information


**Data S1:** Dataset used in the analyses of factors associated with population growth of the goitered gazelle (*Gazella subgutturosa*) across 27 protected areas in Iran.

**Data S2:** ReadMe file describing the dataset, including definitions, descriptions, and measurement units of all variables.

**Table S1:** Growth rates (*r*) and geographic attributes of protected areas in Iran. NP = National Park; WR = Wildlife Refuge; PA = Protected Area; NHA = No‐Hunting Area.
**Table S2:** Component loadings for the principal component analysis of climatic variables.
**Table S3:** Component loadings for the principal component analysis of human land‐use variables.
**Table S4:** Component loadings for the principal component analysis of protection and management variables.

## Data Availability

All the required data are uploaded as Supporting Information.
