# Supplementary material for: Ecological and Management Drivers of Population Fluctuations in the Goitered Gazelle ( Gazella subgutturosa ) Across Iran
Source: Ecol Evol. 2026 Aug 31;16(9):e74234. doi: 10.1002/ece3.74234 (PMC13527858; doi:10.1002/ece3.74234)
Supplement: Supplementary file 1 [file ECE3-16-e74234-s001.docx]

**README and Data Dictionary**

**Dataset title**

Protected-area, climatic, human land-use, and management variables used to analyse population growth of the goitered gazelle (*Gazella subgutturosa*) across Iran

**Dataset description**

This dataset contains site-level data for 27 protected areas in Iran supporting populations of the goitered gazelle (*Gazella subgutturosa*). The dataset was compiled to examine associations between long-term population growth rates and climatic conditions, human land use, and protected-area management characteristics.

The dataset includes the 16-year population growth rate of goitered gazelles for each protected area, together with variables describing protection and management, human land use and anthropogenic pressures, and climatic and vegetation conditions. Most variables represent site-level conditions or long-term averages over the 2007–2022 study period.

The dataset was used for multivariate analyses, including principal component analysis (PCA) and multiple linear regression/General Linear Models (GLMs), to identify major environmental, anthropogenic, and management gradients associated with variation in population growth among protected areas.

**Spatial unit of analysis**

Each row represents one protected area supporting a goitered gazelle population.

**Number of protected areas:** 27

**Country:** Iran

**Study period:** 2007–2022

**Species:** Goitered gazelle (*Gazella subgutturosa*)

**Data dictionary**

1. Site identification and population growth

| Variable | Description | Unit / coding |
| --- | --- | --- |
| NAME | Name of the protected area | Text |
| *r* | Average exponential population growth rate of goitered gazelles over the 2007–2022 study period, estimated as the slope of a linear regression of the natural logarithm of annual population size against year | Annual exponential growth rate |

**Note:** Population counts were obtained from annual direct-count surveys conducted by the Iranian Department of Environment. Counts were treated as indices of relative population size because comparable estimates of detection probability were not available for all protected areas. The variable r therefore represents the estimated long-term growth rate of the monitored population index rather than a detection-corrected demographic growth rate.

**2. Protection and management variables**

| Variable | Description | Unit / calculation |
| --- | --- | --- |
| Plain Area | Area of relatively flat terrain considered potentially suitable for gazelle use, defined as terrain with a slope of less than 30% | ha |
| Edge | Ratio of protected-area perimeter to area | Dimensionless ratio |
| Isolation Index | Mean distance from the focal protected area to the three nearest protected areas supporting goitered gazelles | km |
| Protection Duration | Time elapsed since the initial designation of the protected area | years |
| Protection level | Categorical designation of the protected area converted to an ordinal score representing the level of legal protection | National Park = 8; Wildlife Refuge = 6; Protected Area = 4; No-Hunting Area = 2 |
| guard posts Density | Density of environmental guard posts within the protected area | Number of guard posts per ha |
| Ranger Density | Density of environmental rangers assigned to the protected area | Number of rangers per ha |
| patrol vehicles Density | Density of patrol motorcycles available to environmental rangers | Number of motorcycles per ha |
| Artificial Water Points Density | Density of artificial water points within the protected area | Number per ha |
| Water Source Density | Density of all recorded water sources, including natural and artificial sources, within the protected area | Number per unit area |

**Note:** Protection-level scores were assigned according to the legal designation of each protected area. Higher scores indicate a higher formal level of protection.

**3. Human land-use and anthropogenic pressure variables**

| Variable | Description | Unit / calculation |
| --- | --- | --- |
| Number of Farms | Number of agricultural farms located within the protected area | Count |
| Number of Mines | Number of mining sites located within the protected area | Count |
| Other Human Land Uses | Number of other types of human land use or infrastructure within the protected area, such as police posts, petrol stations, and similar facilities | Count |
| Number of Villages | Number of villages located within the protected area | Count |
| number of Villages in Buffer Zone | Number of villages located within a 15-km buffer surrounding the protected area | Count |
| Number of Wells in Buffer Zone | Number of wells located within a 15-km buffer surrounding the protected area | Count |
| Number of Springs in Buffer Zone | Number of natural springs located within a 15-km buffer surrounding the protected area | Count |
| Number of Cities in Buffer Zone | Number of cities or towns located within a 15-km buffer surrounding the protected area | Count |
| Number of Industrial Facilities in Buffer Zone | Number of industrial facilities located within a 15-km buffer surrounding the protected area | Count |
| Grazing Duration | Duration of the period during which livestock grazing is permitted within the protected area | months per year |
| Paved Road Length | Total length of paved roads within the protected area | km |
| Unpaved Road Length | Total length of unpaved roads within the protected area | km |
| Gazelle Road and poaching Mortality Rate | Mean annual number of recorded goitered gazelle carcasses attributed to road collisions and illegal hunting, expressed relative to the mean annual gazelle population size over the 16-year study period | % |

Buffer-zone definition: For variables referring to the buffer zone, the buffer was defined as a 15-km zone surrounding the boundary of each protected area.

Mortality-rate calculation:

$$Mortality rate=\frac{mean annual number of recorded road and poaching mortalities}{mean annual gazelle population size}\times100$$

This metric is based on recorded mortalities and may therefore underestimate actual mortality where carcass detection or reporting is incomplete.

**4. Climatic and vegetation variables**

Climatic variables represent long-term conditions for the protected areas, primarily calculated over the 2007–2022 study period.

| Variable | Description | Unit |
| --- | --- | --- |
| 16-year average ndvi | Mean NDVI across the 16-year study period, representing average vegetation productivity | NDVI units |
| Spring NDVI change trends | Temporal trend in spring NDVI over the 2007–2022 period | NDVI change per year |
| Average maximum temperature in 6 | Mean maximum temperature for June over the study period | °C |
| Average maximum temperature in 7 | Mean maximum temperature for July over the study period | °C |
| Average maximum temperature in 8 | Mean maximum temperature for August over the study period | °C |
| Average maximum temperature in 9 | Mean maximum temperature for September over the study period | °C |
| Average minimum temperature in 1 | Mean minimum temperature for January over the study period | °C |
| Average minimum temperature in 2 | Mean minimum temperature for February over the study period | °C |
| Average minimum temperature in 12 | Mean minimum temperature for December over the study period | °C |
| Average temperature 16 year | Mean annual temperature averaged over the 16-year study period | °C |
| average rainfall 16-year | Mean annual precipitation averaged over the 16-year study period | mm |
| Average rainfall in 3 | Mean precipitation in March over the study period | mm |
| Average rainfall in 4 | Mean precipitation in April over the study period | mm |
| Average rainfall in 5 | Mean precipitation in May over the study period | mm |
| Average annual number of rainy days 16 year | Mean annual number of days with recorded precipitation over the 16-year study period | days per year |

**NDVI:** Normalised Difference Vegetation Index, used as a remotely sensed indicator of vegetation productivity.

**Data processing and statistical analysi**s

Prior to statistical analysis, numerical variables were standardised where appropriate. Principal Component Analyses were conducted separately for climatic, human land-use, and protection/management variables to reduce dimensionality and summarise correlated predictors into independent multivariate gradients.

A k-means cluster analysis based on long-term climatic variables and seasonal NDVI was used to classify the 27 protected areas into two broad climatic–productivity classes.

Population growth rate (*r*) was used as the response variable in multiple linear regression/General Linear Models. The PCA-derived component scores and protected-area class were considered as explanatory variables. Model simplification and model comparison were conducted using backward elimination and information-theoretic criteria, including AIC and BIC, alongside adjusted R² and ecological interpretability.

The final model retained Protection Component 2 and Human Land-use Component 3 as predictors of population growth.

**Data sources**

Population census data and protected-area management information were obtained from the Iranian Department of Environment. Management-related information was compiled from administrative records and questionnaires completed by protected-area managers.

Climatic and vegetation variables were derived from spatial environmental datasets and remotely sensed NDVI data, as described in the associated manuscript.
