# Supplementary material for: Ecological and Management Drivers of Population Fluctuations in the Goitered Gazelle ( Gazella subgutturosa ) Across Iran
Source: Ecol Evol. 2026 Aug 31;16(9):e74234. doi: 10.1002/ece3.74234 (PMC13527858; doi:10.1002/ece3.74234)
Supplement: Supplementary file 2 [file ECE3-16-e74234-s003.docx]

**Supplementary Material:**

**Table S1.**  Growth rates (*r*) and geographic attributes of protected areas in Iran. NP = National Park; WR = Wildlife Refuge; PA = Protected Area; NHA = No-Hunting Area.

| **Group** | ***r*** | **Plains (%)** | **Area (km²)** | **Longitude (E)** | **Latitude (N)** | **Protected area** |
| --- | --- | --- | --- | --- | --- | --- |
| 2 | 0.0777 | 95 | 2043.4 | 50°10′–50°45′ | 33°20′–33°55′ | Mooteh NP |
| 2 | 0.0051 | 91 | 1138.22 | 49°95′–54°43′ | 36°14′–36°55′ | Ghamishlou NP |
| 2 | 0.0135 | 94 | 507.33 | 51°45′–52°15′ | 32°20′–32°30′ | Kolah-Qazi NP |
| 2 | 0.0448 | 99 | 14 418.19 | 55°00′–57°02′ | 35°00′–36°22′ | Touran NP |
| 1 | −0.0451 | 72 | 486.26 | 52°29′–52°54′ | 29°36′–29°53′ | Bamou NP |
| 2 | 0.1371 | 99 | 846.62 | 56°26′–56°57′ | 36°47′–37°30′ | Miandasht NP |
| 1 | 0.0558 | 90 | 193.34 | 57°03′–57°17′ | 37°07′–37°15′ | Salook NP |
| 1 | 0.0399 | 89 | 916.03 | 53°04′–55°43′ | 37°17′–37°31′ | Golestan NP |
| 2 | −0.0922 | 95 | 224.68 | 57°42′–58°30′ | 36°00′–36°11′ | Shirahmad WR |
| 2 | 0.0581 | 98 | 1560.64 | 57°25′–57°57′ | 33°47′–34°20′ | Robat Shoor WR |
| 2 | 0.0119 | 91 | 1503.89 | 55°14′–55°47′ | 36°37′–37°02′ | Khoshyeylagh WR |
| 1 | 0.1673 | 98 | 35.49 | 45°61′–45°71′ | 34°51′–34°61′ | Gharaviz WR |
| 1 | 0.1746 | 98 | 394.95 | 45°50′–45°62′ | 33°92′–34°12′ | Zale Zard WR |
| 2 | 0.0627 | 89 | 2290.0 | 54°20′–55°15′ | 31°05′–31°35′ | Kalmand PA |
| 2 | 0.1451 | 98 | 543.28 | 60°08′–60°31′ | 33°55′–34°11′ | SirkhooN PA |
| 2 | 0.1485 | 92 | 336.23 | 58°52′–59°08′ | 34°08′–34°22′ | Hengam PA |
| 2 | 0.0399 | 89 | 375.76 | 60°23′–60°50′ | 36°55′–36°65′ | Jangal-e Khajeh PA |
| 1 | 0.0909 | 58 | 71.43 | 58°61′–58°75′ | 35°89′–36°00′ | Raeisi PA |
| 1 | 0.1048 | 97 | 539.9 | 51°06′–51°15′ | 27°05′–28°10′ | Mond PA |
| 1 | −0.0572 | 80 | 1228.07 | 48°28′–48°43′ | 37°03′–37°14′ | Sorkh Abad PA |
| 2 | −0.0333 | 90 | 920.23 | 52°23′–52°53′ | 33°08′–33°30′ | Kahyaz PA |
| 1 | −0.0089 | 75 | 433.24 | 56°08′–56°39′ | 37°20′–37°31′ | Ghorkhud PA |
| 1 | 0.113 | 85 | 253.59 | 49°28′–49°43′ | 36°03′–36°14′ | Bashgol PA |
| 1 | 0.0379 | 15 | 507.14 | 47°09′–48°04′ | 37°01′–37°05′ | Kaghazkonan PA |
| 2 | 0.0898 | 84 | 1878.48 | 52°30′–53°00′ | 30°38′–31°00′ | Basiran NHA |
| 2 | 0.1431 | 100 | 1712.22 | 58°05′–58°52′ | 34°37′–35°05′ | Nobahar NHA |
| 2 | 0.2321 | 83 | 496.46 | 57°06′–57°22′ | 34°09′–34°28′ | Eshgh Abad NHA |

**Table S2.** Component loadings for the principal component analysis of climatic variables

| Variable | PC1 | PC2 | PC3 |
| --- | --- | --- | --- |
| 16-year average NDVI | −0.062 | 0.811 | −0.036 |
| Spring NDVI change trend | 0.327 | 0.437 | 0.696 |
| Average maximum temperature in June | 0.945 | −0.208 | 0.153 |
| Average maximum temperature in July | 0.920 | −0.111 | 0.299 |
| Average maximum temperature in August | 0.915 | 0.096 | 0.347 |
| Average maximum temperature in September | 0.945 | −0.008 | 0.300 |
| Average minimum temperature in January | 0.797 | 0.352 | −0.474 |
| Average minimum temperature in February | 0.897 | 0.167 | −0.375 |
| Average minimum temperature in December | 0.801 | 0.391 | −0.425 |
| 16-year average temperature | 0.978 | −0.024 | −0.175 |
| 16-year average rainfall | 0.166 | 0.958 | 0.034 |
| Average rainfall in March | 0.110 | 0.900 | −0.023 |
| Average rainfall in April | −0.175 | 0.918 | 0.159 |
| Average rainfall in May | −0.571 | 0.670 | 0.009 |
| 16-year average annual number of rainy days | −0.378 | 0.818 | −0.015 |

Values are component loadings from a principal component analysis. Three components were retained based on eigenvalues >1 and inspection of the scree plot. NDVI, Normalised Difference Vegetation Index.

Table S3. Component loadings for the principal component analysis of human land-use variables

| Variable | PC1 | PC2 | PC3 | PC4 |
| --- | --- | --- | --- | --- |
| Number of farms | 0.688 | 0.198 | −0.342 | 0.283 |
| Number of mines | 0.808 | −0.106 | 0.278 | −0.081 |
| Other human land uses | 0.850 | −0.093 | 0.125 | 0.130 |
| Number of villages | 0.597 | 0.566 | −0.383 | −0.027 |
| Number of villages in buffer zone | 0.658 | −0.328 | −0.254 | 0.335 |
| Number of wells in buffer zone | 0.097 | −0.061 | 0.682 | 0.303 |
| Number of springs in buffer zone | 0.396 | 0.213 | 0.610 | 0.342 |
| Number of cities in buffer zone | 0.634 | −0.628 | −0.218 | −0.104 |
| Number of industrial facilities in buffer zone | 0.326 | −0.687 | −0.161 | 0.033 |
| Number of poached gazelle carcasses | 0.815 | −0.421 | 0.119 | −0.018 |
| Grazing duration | 0.460 | 0.569 | −0.150 | 0.302 |
| Paved road length | 0.594 | 0.386 | 0.305 | −0.548 |
| Unpaved road length | 0.633 | 0.124 | 0.034 | −0.639 |
| Gazelle road mortality rate | 0.362 | 0.617 | −0.131 | 0.142 |

Values are component loadings from a principal component analysis. Four components were retained based on eigenvalues >1 and inspection of the scree plot. Buffer-zone variables were measured within 15 km of each protected area.

Table S4. Component loadings for the principal component analysis of protection and management variables

| Variable | PC1 | PC2 | PC3 |
| --- | --- | --- | --- |
| Plain area | −0.392 | 0.208 | 0.472 |
| Edge-to-area ratio | 0.445 | −0.635 | 0.513 |
| Isolation index | 0.354 | −0.257 | −0.073 |
| Protection duration | −0.128 | 0.782 | 0.392 |
| Protection level | 0.052 | 0.672 | 0.600 |
| Guard-post density | 0.672 | 0.541 | −0.451 |
| Ranger density | 0.903 | 0.328 | −0.023 |
| Patrol-vehicle density | 0.858 | 0.379 | −0.229 |
| Artificial-water-point density | 0.882 | −0.232 | 0.287 |
| Water-source density | 0.852 | −0.305 | 0.315 |

Values are component loadings from a principal component analysis. Three components were retained based on eigenvalues >1 and inspection of the scree plot. Plain area refers to the area of terrain with slopes <30%.
